# Leptomeningeal form of Immunoglobulin G4-related hypertrophic meningitis with perivascular spread: a case report and review of the literature

**DOI:** 10.1007/s00234-018-2028-y

**Published:** 2018-05-07

**Authors:** Jasmina Boban, Selin Ardalı, Majda M. Thurnher

**Affiliations:** 1 Faculty of Medicine, Department for Radiology, University of Novi Sad, Hajduk Veljkova 3, Novi Sad, 21000 Serbia; 2 Department of Radiology, Hacettepe University Faculty of Medicine, Sıhhiye, 06100 Ankara, Turkey; 3 Section of Neuroradiology and Musculoskeletal Radiology, Department for Biomedical Imaging and Image-guided Therapy, Medical University of Vienna, Waehringer Guertel 18-20, 1090 Vienna, Austria

**Keywords:** Meningitis, IgG4, Magnetic resonance imaging (MRI)

## Abstract

**Purpose:**

Immunoglobulin G4 (IgG4)-related disease represents a spectrum of fibro-inflammatory disorders that affects various organ systems, including the central nervous system.

**Methods:**

Here we present the case of lgG4-related hypertrophic meningitis with exclusively leptomeningeal involvement and spread via perivascular spaces.

**Results:**

A 58-year-old male patient presented with complex partial seizures. Initial computed tomography examination showed left frontal sulcal hyperdensity. Subsequent magnetic resonance examination revealed FLAIR hyperintensity in the central sulcus, with post-contrast enhancement in the form of “dotted line.” Physical examination, routine laboratory, and cerebrospinal fluid analyses were unremarkable. Meningeal biopsy confirmed IgG4-related meningitis. After corticosteroid treatment, a complete resolution of imaging findings was observed. Two months later, the patient presented with relapsing neurological symptoms and radiological findings in postcentral, precentral, and temporal sulci, resembling the form of “dotted line” contrast enhancement. In addition, linear intraparenchymal enhancement that followed perivascular spaces was seen in the left parietal lobe. After repeated steroid therapy, all lesions resolved completely.

**Conclusion:**

We reported the first case of isolated IgG4-related leptomeningeal involvement with a “dotted line” enhancement and perivascular intraparenchymal spread. Although IgG4-related meningitis represents a rare disease, both clinicians and radiologists should include this condition in the differential diagnosis of unclear leptomeningeal disease.

## Introduction

Immunoglobulin G4 (IgG4)-related disease encompasses a spectrum of fibro-inflammatory disorders characterized by IgG4-positive plasma cell infiltration that can affect almost every organ system [1]. The disease was first described in the pancreas, and then, in the salivary and lacrimal glands, thyroid, kidney, bile ducts, lungs, and retroperitoneum [1, 2]. Histopathologic features include lymphoplasmacytic infiltration of IgG4+ plasma cells, storiform fibrosis, and obliterative phlebitis. Central nervous system (CNS) involvement is relatively rare and mostly seen as hypophysitis. Hypertrophic pachymeningitis is a focal or diffuse thickening of the intracranial and/or spinal meninges and is a recently recognized part of the IgG4-related disease spectrum [3, 4]. Hypertrophic pachymeningitis can be observed in a wide range of diseases, including malignant, immunological, infectious, and vasculitic conditions, and can also be idiopathic. Many of the cases previously described as idiopathic hypertrophic cranial pachymeningitis (IHCPM) might actually belong to the IgG4-related disease spectrum [5].

To date, several cases of pachymeningitis—but only three cases of leptomeningeal involvement in IgG4-related disease have been described [6]. To the best of our knowledge, no case of an isolated leptomeningeal form of IgG4-related disease with perivascular spread has been described. Here, we present the case of histologically proven lgG4-related hypertrophic meningitis with exclusively leptomeningeal involvement and spread via the perivascular spaces.

## Case presentation

**Fig. 1:**
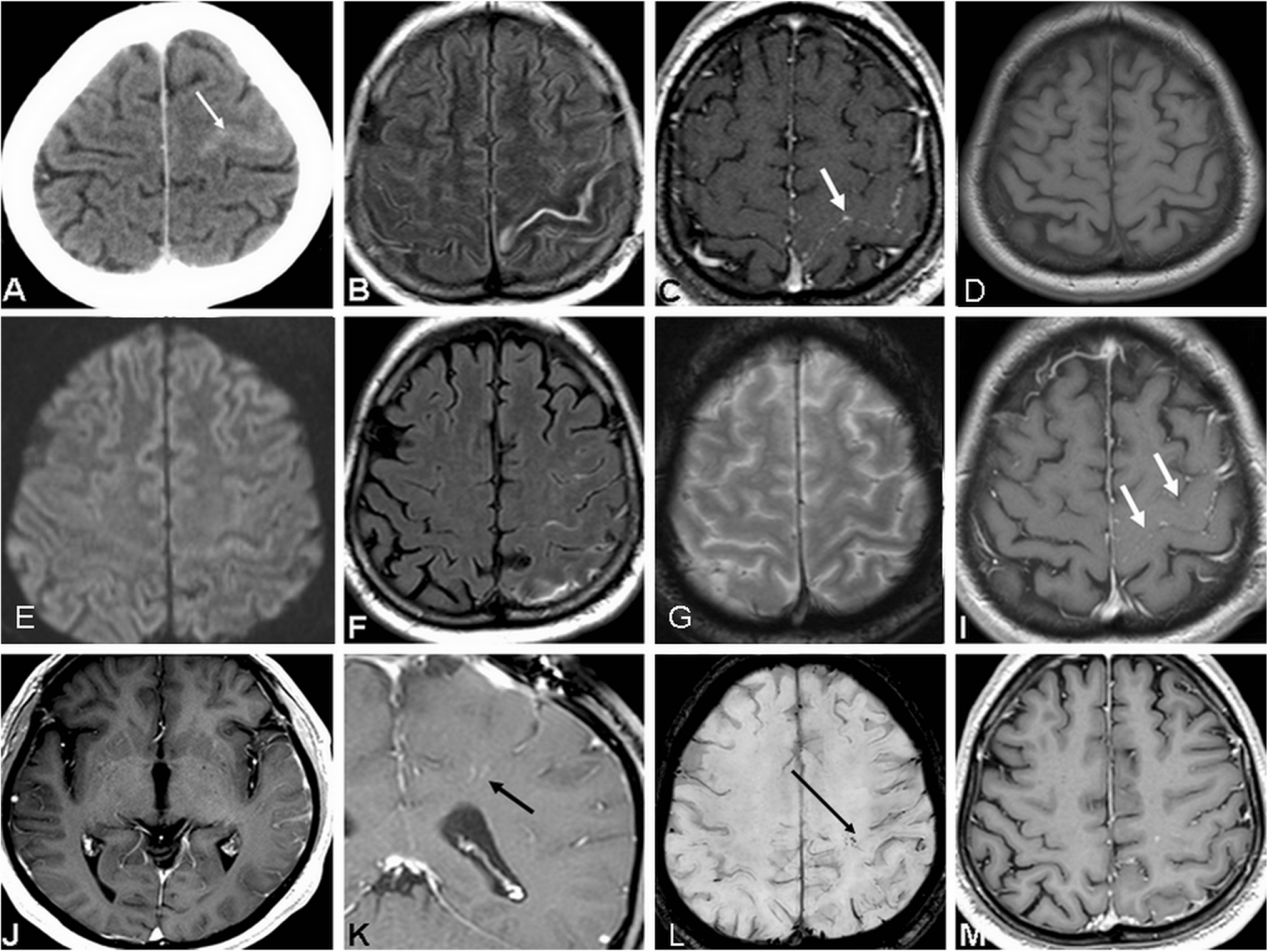
**a**–**m** Axial pre-contrast CT scan showed slightly hyperdense central sulcus (**a**, white arrow). Sulcal hyperintensity was detected on axial FLAIR image (**b**, white arrow). After contrast administration (**c**, white arrow), a leptomeningeal pattern of “dotted line” enhancement was observed, while on native T1W images (**d**) the sulcal narrowing was evident only. There was no restricted diffusion observed (**e**). Relapse occurred after 2 months, affecting postcentral, precentral sulcus (**f**), with no hemosiderin deposits on T2*W images (**g**) and the typical “dotted line” leptomeningeal enhancement (**i**, **j**, white arrows). Spread along perivascular spaces was observed as linear contrast enhancement along dilated veins (**k**, **l**, black arrows). After corticosteroid treatment, 6 weeks later, almost complete resolution was observed (**m**). A discrete dotted micronodular enhancement was still evident in precentral sulcus on the left, as well as along perivascular spaces in the left hemisphere

A 58-year-old previously healthy male patient presented with complex partial seizures. Physical examination and routine laboratory tests were unremarkable. Cerebrospinal fluid (CSF) analysis showed a normal cell count, and normal protein and glucose levels. Initial computed tomography (CT) of the brain showed a subtle sulcal hyperdensity in the left frontal lobe (Fig. 1a). Brain magnetic resonance (MR) imaging revealed a fluid-attenuated inversion recovery (FLAIR) hyperintense left central sulcus (Fig. 1b). On post-contrast T1-weighted images, nodular leptomeningeal enhancement in the form of a “dotted line,” corresponding to the sulcal FLAIR hyperintensity, was observed (Fig. 1c). The underlying gray matter of the central gyrus was FLAIR/T2-weighted hyperintense. On the follow-up MR examination, the enhancement in the central sulcus became thicker and more linear. At this point, a subtle linear enhancement along the perivascular spaces was detected in the underlying white matter of the parietal lobe. The patient underwent surgical biopsy that revealed meningeal deposits of collagenous fibrous tissue, imbued with thick reactive-proliferative, mostly inflammatory infiltrate with numerous plasma cells and monocytes, and several giant round cells. Inflammatory changes were observed along the small blood vessels also, with no signs of obliterative phlebothrombosis. Most plasma cells were positive for IgG4 antibodies on immunohistochemistry, thus indicating the diagnosis of IgG4-related meningitis. Serum IgG and IgG4 levels were within normal range. The workup for immunologic diseases and malignancy was negative. There were no relevant symptoms or findings compatible with the IgG4-related disease spectrum in any other organ systems. After corticosteroid treatment, there was a complete resolution of imaging findings. However, 2 months later, the patient presented with new neurological symptoms (headache, speech difficulties, sleeping disorder). MR revealed FLAIR hyperintensity in the left precentral and postcentral sulci and in the sulci of the left temporal lobe (Fig. 1f–g). The postcentral gyrus appeared hyperintense on FLAIR and T2W. “Dotted line” enhancement, similar to that observed in the initial lesion, was detected in the affected sulci (Fig. 1i, j). In addition, a more prominent, linear intraparenchymal enhancement that followed the perivascular spaces was clearly seen in the left parietal lobe (Fig. 1k, l). New lesions were attributed to the relapse of idiopathic IgG4-related disease. After repeated administration of steroid therapy, lesions resolved almost completely (Fig. 1m). A discrete dotted micronodular enhancement was still evident in precentral sulcus on the left, as well as along perivascular spaces in the left hemisphere.

## Discussion and conclusions

IgG4-related disease is a recently recognized spectrum of complex, immune-mediated, sclerosing inflammatory disorders with common pathologic, clinical, and serologic characteristics [1]. The condition was first described in the pancreas, and then, there were reports of this condition in the orbit, salivary glands, lymph nodes, lungs, kidneys, and other organs [2]. Hypertrophic pachymeningitis is a rare neurologic manifestation of the IgG4-related disease spectrum characterized by localized or diffuse thickening of the dura mater [3, 4]. Recently, IgG4-related pachymeningitis has been suggested to represent some of the cases that were previously diagnosed as idiopathic hypertrophic cranial pachymeningitis (IHCPM) [5].

IgG4-related pachymeningitis generally occurs in men during the fifth and sixth decades. Typical clinical symptoms include headache, seizures or focal deficits, compression or involvement of cranial nerves leading to neuropathies, vessel occlusions, and dural venous sinus occlusions [6]. On CT/MR imaging, hypertrophic pachymeningitis is characterized by diffuse or mass-like thickening of the dura over the cerebral hemispheres and/or the spinal cord [7–9]. Some cases with bulging masses connected to the dura have also been described [9].

To date, to the best of our knowledge, no cases of isolated leptomeningeal IgG4-related meningitis with perivascular spread have been described.

In our patient, an isolated leptomeningeal involvement was present in the frontal, parietal, and temporal sulci. Leptomeningeal enhancement had an interesting pattern of a “dotted line,” consisting of a small nodular thickening of the leptomeninges. Nodular leptomeningeal enhancement can also be observed in malignant or vasculitic diseases [10]. Also, leptomeningeal enhancement was described, although rarely, in some autoimmune disorders. In rheumatoid arthritis, a typical pattern of associated thick dural and leptomeningeal enhancement was observed [11]. U-shaped leptomeningeal enhancement that varied in size, number, and location over time was described in Susac’s syndrome [12]. A recently described entity, Rosai-Dorfman disease, is an idiopathic histiocytic disorder with sinus histiocytosis and massive lymphadenopathy. Several cases with diffuse meningeal involvement on MRI were described, but more commonly in the form of diffuse dural enhancement, such as that in meningioma, lymphoma, or chronic inflammation [13].

Soon after the onset of the leptomeningeal disease, a subtle contrast enhancement was noted in the underlying white matter, along the perivascular spaces, representing inflammatory spread along the perivascular spaces. In addition, T2W/FLAIR signal changes without contrast enhancement were evident in the underlying cortex. These changes probably represented vasogenic edema (no signs of restricted diffusion were present), due to venous congestion and deprived venous outflow. There were no signs of dural involvement or dural sinus thrombosis. Post-contrast T1W images showed enhancement in the narrowed sulci, as in previously described cases of dural thickening [7–9].

There have been only three cases of leptomeningeal involvement in IgG4-related disease reported in the literature [6, 9, 14]. However, in two patients, a history of concomitant rheumatoid arthritis was present; thus, a certain bias in the differential diagnosis was created. One of these two patients also had an adjacent diffuse pachymeningeal enhancement [9]. The third patient was described in a pathologic study that reviewed ten cases of unexplained meningeal pathologic findings, with no imaging or clinical data available. In five cases, after additional immunohistological analysis, IgG4-related disease was confirmed; leptomeningeal involvement was described in one of these cases. Histological findings were similar to those in our case, with a lymphoplasmacytic perivascular inflammatory infiltrate with less prominent sclerosis, and no signs of obliterative phlebitis [6].

Meningeal biopsy is the gold standard for establishing the diagnosis of IgG4-related disease [15]. Diagnostic criteria for IgG4-related pachymeningitis are based on three characteristic histological findings: lymphoplasmacytic infiltration of IgG4-positiveplasma cells, storiform fibrosis, and obliterative phlebitis. In our patient, there was a thick inflammatory infiltrate of plasmacytic and monocytic cells, with the majority of plasma cells positive for IgG4 antibodies. The inflammatory infiltrate was also present along the blood vessels, without signs of extensive fibrosis, concordant with the previous pathologic study [6]. There are no single immunohistochemical features that are pathognomonic for this disease. However, a cutoff value of 10 IgG4 + plasma cells/HPF, and an IgG4+/IgG+ plasma cell ratio > 40%, are recognized as sufficient for the diagnosis of IgG4-related disease of the meninges [15].

CSF analysis is performed to exclude other pathologies, such as CNS infections or malignant disease. Usually, the results of CSF analysis reveal normal glucose levels, normal or slightly elevated protein levels, and a variable degree of pleocytosis (lymphocytic, monocytic) [16]. Results from previous studies have implied moderate to severe damage to the blood-brain barrier, as in other forms of inflammatory meningitis. A higher elevation of CSF IgG4 levels in patients with IgG4-related pachymeningitis when compared with patients with infectious, neoplastic, and inflammatory meningitis has also been reported [16].

Serum IgG4 level may or may not be elevated [17]. In our patient, and in the majority of the cases described in the literature, serum IgG4 levels were within normal ranges.

Although currently there is no consensus about the treatment of IgG4-related pachymeningitis, glucocorticoid therapy is the first choice. Recurrent disease after corticosteroid therapy rarely occurs. However, in our case, recurrence was observed after cessation of steroid therapy after a short period of time, similar to the case described by Shapiro et al. [18]. Cases with recurrent disease are treated with the addition of immunosuppressive, steroid-sparing agents, such as azathioprine, mycophenolate mofetil, methotrexate, and rituximab [1].

In conclusion, this is the first case of isolated IgG4-related leptomeningeal involvement with a “dotted line” enhancement and perivascular intraparenchymal spread. Although a rare disease, both clinicians and radiologists should be aware of this condition as part of the differential diagnosis in patients with leptomeningeal disease. A prompt diagnosis with meningeal biopsy and a specific therapeutic approach might prevent long-term neurological complications.

## Abbreviations

IgG4: Immunoglobulin G4
FLAIR: Fluid attenuation inversion recovery
CNS: Central nervous system
IHCPM: Idiopathic hypertrophic cranial pachymeningitis
CSF: Cerebrospinal fluid
CT: Computed tomography
MR: Magnetic resonance
T1W: T1-weighted
T2W: T2-weighted
